# Association between central sensitization inventory and taste alterations in patients with burning mouth syndrome: a cross-sectional study

**DOI:** 10.22514/jofph.2026.063

**Published:** 2026-09-12

**Authors:** Keita Takizawa, Kana Kawai-Ozasa, Sana Ohnuma, Fu Sakai, Kohei Shimizu, Eli Eliav, Noboru Noma

**Affiliations:** ^1^Department of Oral Medicine, Nihon University School of Dentistry, 101-8310 Tokyo, Japan; ^2^Department of Endodontics, Nihon University School of Dentistry, 101-8310 Tokyo, Japan; ^3^Eastman Institute for Oral Health, University of Rochester Medical Center, Rochester, NY 14620, USA

**Keywords:** Burning mouth syndrome, Central sensitization inventory, Taste alteration

## Abstract

**Background**: Burning mouth syndrome (BMS) is a chronic condition characterized by a burning sensation in the mouth without observable lesions, lasting over two hours daily for more than three months. In this study, we aimed to examine whether central sensitization inventory (CSI) scores differ between primary and secondary BMS (SBMS), arising from identifiable causes, and to explore their associations with taste thresholds and pain characteristics. **Methods**: This study was conducted between February 2022 and September 2025 and included 30 patients with BMS, 30 patients with SBMS, and 30 healthy controls. Taste assessments, including sweet, salty, sour, and bitter solutions and CSI evaluations, were performed. Statistical analyses, including correlation and comparison tests, were performed using the SPSS software. **Results**: Patients with BMS exhibited significantly larger pain visual analog scale (VAS) and CSI scores than those with SBMS. There were no significant differences in taste perception thresholds for the four flavors among the three groups. CSI scores showed significant positive correlations with taste thresholds for sweetness, saltiness, and bitterness in the BMS group, whereas no such correlations were observed in the SBMS group. The pain VAS scores in the BMS group were positively correlated with age and CSI scores. Receiver operating characteristic analysis revealed acceptable CSI discrimination for distinguishing BMS from SBMS (Area under the curve = 0.76). A cutoff of 27 yielded 70% sensitivity and 77% specificity, with an overall accuracy of 73%. **Conclusions**: Although no significant differences in taste thresholds were observed among the three groups, significant associations between the CSI scores and taste thresholds were identified exclusively in patients with BMS. While CSI scores do not directly assess central sensitization, they might reflect clinical features associated with central sensitization-related symptom burden in BMS, as observed for taste thresholds, pain intensity, and age. These findings suggest that taste alterations in BMS might be associated with central sensitization-related clinical features.

## 1. Introduction

Burning mouth syndrome (BMS) is a persistent condition characterized by the International Classification of Orofacial Pain (ICOP) as “a sensation of burning or discomfort within the mouth that occurs regularly for over 2 hours daily for more than 3 months without observable causative lesions upon clinical examination and investigation” [1]. The mechanisms underlying BMS have not been fully elucidated; however, physiological and psychological factors have been implicated [2, 3, 4]. Pain associated with BMS does not change with eating or drinking, and some patients experience pain relief with sweet foods [5, 6]. Additionally, individuals with BMS may report subjective changes in somatosensory perception, such as alterations in taste, phantom taste sensations, reduced saliva production, and mood disturbances [7].

Alternatively, a burning sensation in the mouth and surrounding areas can result from various factors, including localized causes (such as poorly fitting dentures) or systemic and local diseases. Such instances are classified as secondary BMS (SBMS) and are distinct from primary BMS [8]. Local factors and diseases that trigger SBMS include oral candidiasis, galvanism, dermatological conditions such as lichen planus, allergies, reduced saliva production (hyposalivation), and dry mouth (xerostomia). Systemic disorders that contribute to SBMS include hormonal fluctuations, nutritional deficiencies (vitamin B12, folic acid, or iron deficiencies), diabetes mellitus, emotional stress, and the use of certain medications, either directly or indirectly. Addressing the underlying conditions typically relieves the burning sensation experienced by patients with SBMS [1].

Over the past two decades, studies of patients with BMS have shifted our understanding from idiopathic to neuropathic, involving both the peripheral and central nervous systems (CNS). Neuropathic origins include small fiber neuropathy, subclinical pathology of the oral system, or abnormal pain processing in the CNS [9, 10]. Quantitative sensory testing has confirmed sensory profile variations, including hypoesthesia or gain-of-function signs, in thermal modalities [10]. Another hypothesis relates BMS symptoms to damage to the gustatory system, including the chorda tympani. Chorda tympani dysfunction and the loss of central inhibitory mechanisms may lead to burning sensations in patients with BMS. Studies have shown that, in some patients, chorda tympani dysfunction assessed using quantitative sensory testing is associated with elevated taste detection thresholds and bilateral symptoms, even in cases of unilateral dysfunction, suggesting possible involvement of CNS mechanisms [7, 8].

The International Association for the Study of Pain defines central sensitization (CS) as the heightened responsiveness of CNS neurons to normal or subthreshold input [11]. CS is important in conditions such as osteoarthritis, shoulder pain, whiplash injury, fibromyalgia, and tendinopathy. The CS inventory (CSI) screens for CS-related symptoms across various syndromes. It consists of 25 items that measure physical, emotional, head/jaw, and urological symptoms. Confirmatory factor analysis supported this bi-factor model. Higher CSI scores correlate with chronic pain and predict poor postoperative outcomes, possibly owing to increased brain-derived neurotrophic factor levels that contribute to CS [12, 13].

In this study, we aimed to evaluate whether CSI scores differ between BMS and SBMS and to explore their clinical associations with taste thresholds, age, pain intensity, and pain duration. We hypothesized that patients with BMS would exhibit higher CSI scores and pain intensity than those with SBMS.

## 2. Materials and methods

This study was approved by the Ethics Committee of Nihon University School of Dentistry (EP16 D020-1) and conducted in accordance with the Declaration of Helsinki. This study conformed to the strengthening the reporting of observational studies in epidemiology (STROBE) guidelines. Informed consent was obtained from all the patients and volunteers.

### 2.1 Participants

The recruitment and data collection period was between February 2022 and September 2025.

This study included 30 females with BMS, 30 with SBMS, and 30 healthy female volunteers. None of the participants had a documented history of psychiatric, neurological, or chronic pain disorders, nor had they undergone dental procedures in the six months preceding the study, except for routine periodontal maintenance. Patients were classified into the BMS group and the SBMS group based on the ICOP diagnostic criteria. Specifically, patients complaining of a burning sensation in the mouth were, on one hand, classified into the SBMS group if systemic factors (such as anemia, vitamin or folate, iron, or zinc deficiency, Sjögren’s syndrome, diabetes, or hypothyroidism) or local factors (such as oral candidiasis, oral lichen planus, reduced salivary flow, or metal allergies) were present. According to the ICOP, SBMS is classified as gingival pain or oral mucosal pain. On the other hand, patients who reported a burning sensation despite the absence of identifiable local or systemic conditions, and whose results from blood tests, bacterial culture tests, cytology, and salivary flow rate were normal, were classified into the BMS group. All patients were assessed at their initial visit, and none of them had received any prior BMS treatment.

The examinations were conducted in a tranquil room at a controlled temperature of 20–23 °C. While the recruiting researcher (NN) was informed of each participant’s status, the examiner (KT) was blinded to this information. Each participant underwent taste evaluation after completing CSI. Patients diagnosed with BMS or SBMS were evaluated for spontaneous intraoral pain using a visual analog scale (VAS), defined as 0 on one end and 100 on the other.

### 2.2 CSI

The CSI consists of two components, A and B. Part A is a 25-item self-report questionnaire that evaluates common health-related symptoms associated with central sensitivity syndromes (CSSs). The questionnaire consisted of 25 questions regarding health-related symptoms categorized into four factors: physical symptoms, emotional distress, headache/jaw symptoms, and urological symptoms [14]. Participants rated each item on a 5-point Likert scale ranging from 0 (never) to 4 (always), resulting in total scores ranging from 0 to 100. Although not scored, Part B identified whether specific disorders, including the seven distinct CSSs, were previously diagnosed. These include restless legs syndrome, chronic fatigue syndrome, fibromyalgia, temporomandibular joint disorder (TMD), migraine or tension headaches, irritable bowel syndrome, multiple chemical sensitivities, neck injuries (including whiplash), anxiety, panic attacks, and depression.

Recently, five severity levels (subclinical, 0–29; mild, 30–39; moderate, 40–49; severe, 50–59; and extreme, 60–100) have been defined to aid in the clinical interpretation of CSI [15]. These severity levels offer a comprehensive framework for clinicians to evaluate the severity of CSSs [15].

### 2.3 Taste assessment

Taste was assessed using the whole-mouth method. Taste solutions consisted of sucrose (sweet), sodium chloride (salty), tartaric acid (sour), and quinine hydrochloride (bitter) at five concentrations (1–5) (Table 1). The participants were instructed to protrude their tongues forward and tilt their heads slightly upward. The tip of the taste disk container was placed approximately 5 cm above the anterior tongue to prevent direct contact. One drop of the taste solution was dispensed onto the anterior midline of the tongue. The participants held the solution in their mouths before selecting a response from the taste quality indication chart using their fingers. The presentation of the taste solutions proceeded from low to high concentrations, and when the taste qualities changed, the solutions were rinsed with distilled water. The minimum concentration at which a correct response was obtained for each taste quality was considered the recognition threshold.

**Table 1.** Ingredients for each taste.

Taste thresholds	Ingredient	1	2	3	4	5
Sweetness	Sucrose	15 mg (0.3%)	125 mg (2.5%)	500 mg (10%)	1000 mg (20%)	4000 mg (80%)
Saltiness	Sodium chloride	15 mg (0.3%)	62.5 mg (1.25%)	250 mg (5%)	500 mg (10%)	1000 mg (20%)
Sourness	Tartaric acid	1 mg (0.02%)	10 mg (0.2%)	100 mg (2%)	200 mg (4%)	400 mg (8%)
Bitterness	Quinine hydrochloride	0.05 mg (0.001%)	1 mg (0.02%)	5 mg (0.1%)	25 mg (0.5%)	200 mg (4%)

### 2.4 Sample size

Sample size calculations were performed using the G*Power software (version 3.1.9.7, Heinrich Heine University Düsseldorf, Düsseldorf, NRW, Germany). One-way analysis of variance (ANOVA) was used to detect differences between the BMS, SBMS, and control groups. The significance level was set at α = 0.05, with a statistical power of 0.80 and a medium effect size (*f* = 0.40). The minimum required sample size was 66 (22 per group).

### 2.5 Statistical analyses

The normality of the outcome distribution was assessed using the Shapiro-Wilk test. Data are presented as the mean and standard deviation (SD) for parametric data, median and interquartile range (IQR) for nonparametric data, and box-and-whisker plots for figures. Because the age and CSI score data were normally distributed, they were presented as mean and standard deviation, and a one-way ANOVA was used. Upon confirming a significant overall effect, Tukey’s honest significant difference test was performed to adjust for multiple comparisons. The other outcome measures showed significant deviations from normal distribution, and consequently, non-parametric tests were selected for analysis. The VAS and pain duration of the BMS and SBMS groups were analyzed using the Mann-Whitney U test. Taste solutions (sweet, salty, sour, and bitter) were analyzed using the Kruskal-Wallis test. When a significant difference was detected, *post-hoc* pairwise comparisons were performed using the Bonferroni correction. Spearman’s correlation analysis was used to evaluate possible relationships among the outcomes. Receiver operating characteristic (ROC) curve analysis was performed to evaluate whether the CSI could discriminate between BMS and SBMS. The area under the curve (AUC) was calculated, and the optimal cutoff value was determined using the Youden index. Furthermore, we conducted an additional subgroup analysis by stratifying the SBMS group into two subcategories: oral lichen planus (OLP) and non-OLP conditions (including candidiasis, Sjögren’s syndrome, pemphigus/pemphigoid, and other etiologies). SPSS software (version 30.0; IBM Corp., Armonk, NY, USA) was used for all the statistical analyses. Statistical significance was set at *p *< 0.05.

## 3. Results

Table 2 presents the mean, standard deviation, median, and interquartile range for each evaluation.

**Table 2.** Psychophysical profiles, the CSI scores, and taste thresholds in three groups.

		Control group	BMS group	SBMS group
Age (yr)		60.5 ± 9.0	62.5 ± 12.2	61.8 ± 13.0
VAS score		-	40.0 (30.0–70.0)	20.0 (7.75–23.75)
Pain duration (mon)		-	12.0 (3.0–36.0)	20.0 (3.0–60.0)
CSI score		18.3 ± 8.9	35.1 ± 15.7	20.7 ± 11.5
Taste thresholds				
	Sweetness	2 (2–3)	3 (2–3)	2 (2–3)
	Saltiness	2 (2–3)	3 (2–3)	2 (2–3)
	Sourness	3 (2–3)	3 (2.25–3)	3 (2–3)
	Bitterness	2 (2–2)	3 (2–3)	2 (2–3)

*VAS: visual analog scale; CSI: central sensitization inventory; BMS: burning mouth syndrome; SBMS: secondary burning mouth syndrome.*

The average ages in the Control group, BMS group, and SBMS group were 60.5 ± 9.0 years, 62.5 ± 12.2 years, and 61.8 ± 13.0 years, respectively. ANOVA showed no significant differences in age among the three groups (*p *= 0.89). The median and IQR of VAS scores in the BMS group, 40.0 (30.0–70.0) mm, were significantly higher than those of the SBMS group, 20.0 (7.75–23.75) mm (Fig. 1).

**Fig. 1.** Significant difference in VAS between the BMS group and the SBMS group. VAS scores in the BMS group, 40.0 (30.0–70.0) mm, were significantly higher than those of the SBMS group, 20.0 (7.75–23.75) mm. Data are presented as median and interquartile range (IQR). ***p < 0.001. VAS: visual analog scale; BMS: burning mouth syndrome; SBMS: secondary burning mouth syndrome.

The median and IQR of pain duration in the BMS group and SBMS group were 12.0 (3.0–36.0) months and 20.0 (3.0–60.0) months, respectively. There were no significant differences between the BMS and SBMS groups (*p *= 0.91). The mean and SD of CSI scores in the Control group, BMS group, and SBMS group were 18.3 ± 8.9, 35.1 ± 15.7, and 20.7 ± 11.5, respectively. The BMS group showed significantly higher CSI scores than those of the control and SBMS groups (Fig. 2).

**Fig. 2.** Significant differences in CSI scores among groups. The Control group, BMS group, and SBMS group were 18.3 ± 8.9, 35.1 ± 15.7, and 20.7 ± 11.5, respectively. The BMS group showed significantly higher CSI scores than those of the control and SBMS groups. Data are presented as mean and standard deviation (SD). ***p < 0.001. CSI: central sensitization inventory; BMS: burning mouth syndrome; SBMS: secondary burning mouth syndrome.

No significant differences in the basic taste thresholds for the four tastes were observed among the BMS, SBMS, and control groups. Spearman’s correlation analyses were performed for the following variables: age, pain duration, CSI scores, and taste solutions (sweetness, saltiness, sourness, and bitterness). In the BMS group, positive correlations were found between CSI scores and saltiness and bitterness, with a weak positive correlation between CSI scores and sweetness (Fig. 3). However, no correlation was found between CSI scores and sourness. In addition, in the SBMS and control groups, no correlations were found between CSI scores and the four tastes. In the BMS group, the VAS scores were positively correlated with age and CSI scores (Fig. 4). Moreover, a weak positive correlation was observed between VAS scores and bitterness. In the SBMS group, no correlations were observed between other assessments. In the Control group, a negative correlation was observed between age and bitterness; however, similar to the SBMS group, no correlations were observed between the other assessments. The ROC analysis indicated moderate discriminative ability, with an area under the curve (AUC) of 0.76. An optimal cutoff value of 27, identified using the Youden index, provided a sensitivity of 70% and a specificity of 77% (Fig. 5).

**Fig. 3.** Correlation between CSI scores and the four tastes. In the BMS group, Spearman’s rank correlation of CSI scores *vs.* sweetness threshold (a), saltiness threshold (b), sourness threshold (c), and bitterness threshold (d). A positive correlation was observed between CSI score and sweetness, saltiness, and bitterness, but no correlation was observed with sourness. Spearman’s correlation coefficient (*r*) ranges between +1 and −1. CSI: central sensitization inventory.

**Fig. 4.** Correlation between VAS and age and CSI scores in the BMS group. In the BMS group, Spearman’s rank correlation of VAS scores *vs.* CSI scores (a) and age (b). A positive correlation was observed between VAS and age, as well as between VAS and CSI scores. Spearman’s correlation coefficient (*r*) ranges between +1 and −1. VAS: visual analog scale; CSI: central sensitization inventory.

**Fig. 5.** ROC curve for the CSI in distinguishing BMS from secondary SBMS. The ROC curve demonstrates acceptable discriminative performance, with an area under the curve (AUC) of 0.76. The optimal cutoff value of 27, determined using the Youden index, yielded a sensitivity and specificity of 70% and 77%, respectively.

Secondary BMS was attributed to identifiable local or systemic conditions, including oral lichen planus (n = 19), oral candidiasis (n = 5), Sjögren’s syndrome (n = 2), pemphigus/pemphigoid (n = 1), and others (n = 3). *Post hoc* pairwise comparisons using Dunn’s test with Holm correction revealed that CSI scores were significantly higher in the BMS group compared with the OLP (*p* = 0.013) and non-OLP (*p* = 0.004) groups. No significant difference was observed between the OLP and non-OLP groups (*p* = 0.33).

## 4. Discussion

CS refers to the activity-dependent amplification of neural signaling within the CNS, resulting in pain hypersensitivity, even in the absence of ongoing peripheral nociceptive input [16]. The neurophysiological mechanisms underlying CS, in a broad sense, involve CNS modulation, such as synaptic plastic changes, including N-Methyl-D-aspartic acid (NMDA) receptor activation and the attenuation of descending pain inhibitory pathways. This results in increased pain intensity and the expansion of neuronal receptive fields in the spinal cord and brainstem, leading to widespread pain [17, 18].

CSSs have been proposed to be related to the pathophysiology of chronic pain, including conditions such as fibromyalgia, irritable bowel syndrome, TMD, migraine, tension-type headache, and chronic pelvic pain disorder [17, 18, 19]. Neurophysiological techniques have demonstrated enhanced ascending pain pathways and weakened descending pain-inhibiting pathways in patients with these disorders. Furthermore, such dysregulation has also been noted in chronic pain conditions within the orofacial pain domain, such as BMS and TMD [20, 21]. Some of these unexplained refractory symptoms may be accompanied by CNS dysregulation, involving not only painful physical symptoms but also non-painful physical symptoms, such as fatigue, sleep disorders, and sensory hypersensitivity, as well as psychological symptoms, such as anxiety and impaired concentration [22].

The significantly higher CSI scores observed in patients with BMS, together with their positive correlation with VAS scores, support the presence of clinical features associated with CS-related symptom burden. These findings are consistent with previous reports demonstrating altered central pain modulation in BMS, including impaired conditioned pain modulation and altered sensory processing [7, 9, 23]. Neuroimaging studies have further demonstrated altered brain network connectivity and structural changes, including abnormalities in prefrontal regions, thereby supporting the involvement of central pain processing in this condition [24]. However, notably, previous studies have reported only limited and inconsistent associations between the CSI and quantitative sensory testing, indicating that CSI might reflect subjective symptom burden rather than objective neurophysiological sensitization [25]. In addition, the BMS group showed a positive correlation with age and VAS score. However, reports on the correlation between age and VAS scores are not necessarily consistent [26, 27]. Therefore, further analyses considering the country and social background of each disease, as well as examining the relationship with age groups individually, are necessary. The present findings suggest that the CSI has acceptable, albeit limited, discriminative ability for distinguishing BMS from SBMS (AUC = 0.76). Using a cutoff value of 27, the sensitivity (70%) and specificity (77%) indicate a reasonable balance between case detection and exclusion; however, the overall accuracy (73%) suggests that CSI alone is insufficient as a definitive diagnostic tool. Rather, it may serve as a complementary measure within a broader clinical assessment framework.

Taste disturbances, such as changes or distortions in taste perception, persistently altered taste sensations (dysgeusia), and phantom tastes, are commonly experienced by individuals with BMS. Studies have reported taste disturbances in 11–69% of patients with BMS [28, 29]. Among these patients, 67–88% reported experiencing phantom tastes, whereas 59–67% reported dysgeusia [28, 30]. Phantom tastes reported by patients with BMS include bitterness, metallic taste, or a combination of both [28, 30]. Alterations in the perception of salty, sweet, sour, and bitter tastes have also been observed. For instance, sour and bitter tastes may be perceived as stronger, sweet tastes as weaker, and salty tastes as stronger or weaker than usual [28].

Psychophysical research on taste perception thresholds and intensity scaling in individuals with BMS supports clinical findings of subjective taste disturbances. In patients with BMS, thresholds for detecting sweet, salty, and bitter tastes have been observed to be significantly higher than those in the Control group [31, 32]. Individuals also experience difficulties identifying and recalling taste qualities [29]. A study using taste strips revealed that patients with BMS were notably less sensitive than controls to various taste solutions, including sucrose, citric acid, sodium chloride, and quinine hydrochloride dissolved in water [33]. Imura *et al*. [34] reported that taste recognition thresholds for sweet, salty, umami, and bitter tastes were unaffected in the BMS group. However, the taste threshold for sourness was significantly higher in the BMS group.

Several studies utilizing electrogustometry to assess electrically induced taste and somatosensory tingling thresholds of the tongue have revealed diminished function of the taste system in primary BMS, providing evidence supporting the notion that BMS is associated with taste and chorda tympani dysfunctions. An initial study by Eliav *et al*. [8] compared the electrical taste and tingling thresholds of patients with BMS, individuals with secondary burning-mouth symptoms, and healthy controls. Both taste detection thresholds and the mean electrical taste/tingling threshold ratio were notably higher in patients with BMS than in the other two groups. Despite considerable variability in taste detection thresholds among the participants, the authors advocated the use of the electrical taste/tingling detection threshold ratio as a sensitive clinical diagnostic tool. Furthermore, their findings suggested that unilateral chorda tympani hypofunction is adequate to induce bilateral burning sensations, potentially due to CS processes. Similarly, Nasri-Heir *et al*. [35] observed a significantly higher electric taste/tingling detection threshold ratio in patients with BMS than in controls, with the disparity being more pronounced in patients with longer BMS duration. Additionally, Gremeau-Richard *et al*. [36] and Just *et al*. [33] reported increased electrical taste thresholds in patients with BMS. Moreover, a later study suggested decreased pain sensitivity in patients with BMS, as evidenced by the elevated detection threshold for capsaicin irritation in patients with BMS compared with controls [33].

In this study, no significant differences in the perception of sweet, salty, sour, and bitter flavors were observed among the BMS, SBMS, and control groups. We observed that higher CSI scores were positively associated with higher detection thresholds for salty and bitter stimuli and weakly positively associated with sweet stimuli in the BMS group. However, there was no correlation between CSI and taste thresholds in the SBMS and Control groups.

To the best of our knowledge, no previous studies have examined the relationship between taste thresholds and CSI scores. Our results suggest that taste disturbance might be associated with clinical features related to CS in patients with BMS. Previous studies have suggested that in BMS, reduced afferent input from the chorda tympani nerve leads to desensitization of the pain-transmitting trigeminal nerve, thereby amplifying tongue pain [8, 35]. Although no significant group differences in taste thresholds were observed, the presence of significant correlations between CSI scores and taste thresholds exclusively in the BMS group suggests that taste alterations in the BMS group might be associated with CS-related clinical features, rather than peripheral gustatory dysfunction alone [30, 33].

Interestingly, no correlation was observed between CSI scores and sour taste thresholds in the BMS group. Sour taste perception is primarily mediated by proton (H^+^) ions, which can directly activate acid-sensitive ion channels and nociceptive afferents on the tongue. Therefore, local pH-related stimulation of trigeminal nociceptors may be less influenced by CS-related mechanisms, making sour taste perception less sensitive to such influences [31, 37].

This study retains several limitations. First, given the limited sample size, the findings of this study should be interpreted as preliminary. Second, the SBMS group comprised heterogeneous etiologies, including oral lichen planus, candidiasis, and autoimmune diseases, potentially differing in pain characteristics and oral manifestations, thereby limiting interpretability and generalizability. To address this concern, we performed a subgroup analysis stratifying SBMS into OLP and non-OLP groups. *Post hoc* Dunn’s test with Holm correction demonstrated that CSI scores were significantly higher in the BMS group compared with both the OLP (*p* = 0.013) and non-OLP (*p* = 0.004) groups, whereas no significant difference was observed between the OLP and non-OLP groups (*p* = 0.33). Nevertheless, the heterogeneity within SBMS might not have been fully captured. In addition, all participants were Japanese, thereby potentially limiting generalizability across different racial and ethnic populations. Future studies in more diverse cohorts are warranted. Third, the whole-mouth recognition threshold approach employed in this study might have limited sensitivity for detecting subtle or region-specific taste dysfunction compared with more localized or quantitative methods, such as electrogustometry. This methodological constraint could, at least in part, account for the absence of significant between-group differences in taste thresholds observed herein, despite prior evidence indicating altered taste function in BMS.

Future studies should also examine changes in taste thresholds across different CSI severity levels and investigate the activity in pain-taste-related brain regions using functional Magnetic Resonance Imaging (fMRI).

## 5. Conclusions

While CSI scores could not directly assess CS, they might reflect clinical features associated with CS-related symptom burden in BMS. Moreover, the positive correlations between CSI scores and taste thresholds observed in patients with BMS suggest a potential association with CS-related clinical characteristics. Furthermore, these findings indicate that age and CS-related symptom burden might influence pain intensity as measured by VAS.
